# Web-based interrogation of gene expression signatures using EXALT

**DOI:** 10.1186/1471-2105-10-420

**Published:** 2009-12-14

**Authors:** Jun Wu, Qingchao Qiu, Lu Xie, Joseph Fullerton, Jian Yu, Yu Shyr, Alfred L George, Yajun Yi

**Affiliations:** 1 Translational Medicine Group, Shanghai Center for Bioinformation Technology, Shanghai, 200235, China; 2 Department of Medicine, Vanderbilt University, Nashville, TN 37232-0275, USA; 3 Cancer Research Institute and Human Morphology Center, University of South China, Hengyang, 421001, China; 4 Department of Biostatistics, Vanderbilt University, Nashville, TN 37232-0275, USA; 5 Institute for Integrative Genomics, Vanderbilt University, Nashville, TN 37232-0275, USA

## Abstract

**Background:**

Widespread use of high-throughput techniques such as microarrays to monitor gene expression levels has resulted in an explosive growth of data sets in public domains. Integration and exploration of these complex and heterogeneous data have become a major challenge.

**Results:**

The EXALT (**EX**pression signature **A**na**L**ysis **T**ool) online program enables meta-analysis of gene expression profiles derived from publically accessible sources. Searches can be executed online against two large databases currently containing more than 28,000 gene expression signatures derived from GEO (Gene Expression Omnibus) and published expression profiles of human cancer. Comparisons among gene expression signatures can be performed with homology analysis and co-expression analysis. Results can be visualized instantly in a plot or a heat map. Three typical use cases are illustrated.

**Conclusions:**

The EXALT online program is uniquely suited for discovering relationships among transcriptional profiles and searching gene expression patterns derived from diverse physiological and pathological settings. The EXALT online program is freely available for non-commercial users from http://seq.mc.vanderbilt.edu/exalt/.

## Background

Microarray technology enables simultaneous measurements of thousands of gene expression levels in parallel. An overwhelming amount of gene expression data has been generated across a wide spectrum of diseases and experimental conditions. The public repositories, Gene Expression Omnibus (GEO) at the National Center for Biotechnology Information (NCBI) [1] and ArrayExpress at the European Bioinformatics Institute[2], have been established to store microarray data sets. However, these data are neither intuitively meaningful nor directly comparable. A critical challenge is how to utilize these data sets to maximize the information gained.

Efforts have been made to improve microarray data utilization by providing summarized microarray results. As examples, GEO provides gene expression profiles for a given data set, while Oncomine computes significant gene lists from published cancer studies based on common experimental design [3]. A specific pattern or signature of gene expression deduced from a set of differentially expressed genes can be a powerful biomarker. This can be used for determining drug responses, clinical outcomes, and disease diagnosis such as cancer, organ transplant rejection, diabetes, and viral infection [4-9]. Indeed for some diseases, ascertainment of gene expression signatures is rapidly becoming the standard of care for determining disease prognosis and formulating personalized therapy [10].

However with the GEO website, a user can only compare gene expression levels within a given data set. Oncomine makes meta-analysis available by offering pre-processed signatures from published cancer studies, but data sets in Oncomine can only be queried by gene name or experimental keyword rather than by a signature. All profile analysis reports in Oncomine are based on sample groups and significant genes within the selected data set. Hence, search entries in GEO or Oncomine are limited to meta-data attributes from experiment descriptions, sample information, and probe IDs rather than the expression data themselves. Neither system supports a signature comparison or a signature similarity search across all data sets. It remains difficult to ask targeted biological questions at the signature level by querying microarray results across a large collection of data sets.

We previously described a bioinformatic strategy (**EXALT**) to enable comparisons of microarray data across experimental platforms, different laboratories, and multiple species [11]. EXALT uses gene expression signatures extracted from expression values to query a large formatted collection of microarray results. We accomplished this by first transforming a large collection of gene expression data into a rank ordered format of differentially expressed gene signatures within each experiment. EXALT offers an efficient and cost-effective way to discover intrinsic similarities and relationships among gene expression profiles.

We have now implemented a web-enabled version of EXALT (EXALT online) with enhanced functionality and ready access to two expansive databases of gene expression signatures (currently > 28,000). The gene expression signatures are derived from GEO and published transcriptional profiling studies of human cancer. The EXALT online program provides an easy-to-use web-interface for executing multiple query types to search for homologous signatures online - a feature not offered by any other currently available web-based tools.

## Implementation

The EXALT online program organizes genes exhibiting significant differences in expression within an experiment in a special format called a "triplet". A "triplet" includes a gene identifier, a direction of differential expression, and a confidence level called Q score. All signatures are extracted using the same statistical method [11]. A signature defined by the EXALT online program is a set of significant genes with their corresponding statistical scores and direction codes.

## Data sources

GEO GDS sets were processed into signatures in batch mode using EXALT. Published human cancer expression profiling studies were identified in PubMed by a search strategy using keywords "human", "cancer", and "microarray". From a total of 1,062 publications, 310 studies had publically available data that could be manually curated.

## Signature extraction

A 4-step process was used to extract gene expression signatures from individual human cancer data sets. First, data were manually formatted to a common data type. Second, we tested each gene for significant differential expression using a common statistical method. The P-values determined for each significant gene were adjusted by the false discovery rate (FDR) method using Q values [12]. Q values were then converted to the logarithm of the reciprocal for the Q scores (-log[Q value]). Third, a list of significant genes with a Q value ≤ 0.2 was produced. Finally, gene expression signatures were encoded in the form of a list of "triplets". A triplet format was defined as a Gene symbol - Direction Code - Q score. The direction code was determined by the relative difference between two group means and could have one of three values (U, up; D, down; X, uncertain). Signatures were stored in a relational database linked with clinical outcome data and experimental features from the original study.

## Signature databases

Gene expression signatures derived from GEO and published human cancer studies were stored in two separate relational signature databases, i.e. GEO signature database (SigDB) and human cancer SigDB (HuCaSigDB). The current databases are limited to samples derived from human, mouse, and rat. They contain 25,613 signatures derived from 1,445 data sets in GEO and 2,391 human cancer signatures from 532 data sets from published human cancer studies. The current version of HuCaSigDB is highly enriched in studies of human breast cancer.

## Signature comparison

The EXALT online program enables comparisons of gene expression signatures in both GEO SigDB and HuCaSigDB using the same algorithms as we described before [11]. Data access can be initiated from multiple search entries. During a search for homologous signatures, query genes are compared to all subject signature genes in the database. The similarity between a query signature and a subject signature is first determined by gene symbol match and concordance in the direction of differential expression. Then, a normalized total identity score is calculated based on Q scores from the query and the subject signature. The significance level of the similarity is determined by a simulation analysis [11]. Significant matching results between a query and subject signatures are displayed graphically. When users enter a single gene or a set of gene symbols for co-expression analysis, a regular database search is performed based on query gene symbols.

## System architecture

The EXALT online program was implemented using Microsoft .Net technology with typical three-tiered architecture. A standalone web server is in charge of user query data processing and result presentation while an independent application server provides web services for data analysis and query process. A relational database management system (RDBMS) manages signature data storage and retrieval. This type of architecture allows for concurrent and remote access to many users in a scalable and reliable way.

The implementation of web application logic and user interfaces was achieved by using ASP.NET 2.0, which is an integral part of the .NET Framework 2.0. ASP.NET was chosen because of its productivity gains and powerful libraries for image processing and database access. The data query and analysis algorithms were implemented by internet application programming interfaces (web service API). The web services can be accessed over a network and executed on a remote application server hosting the requested services. The EXALT online program application has been successfully tested on internet browsers from Microsoft Window, Mac OS X, and Linux X Window.

## Results

The EXALT online program interface facilitates the uploading of user data, database searching, and comparing signatures. We previously described the user data uploading process and a general strategy to compare gene expression signatures among microarray data sets [11]. The new EXALT online program enables online keyword searching, signature homology analysis, and gene co-expression analysis. Detailed instructions for using the EXALT online program features can be found at: http://seq.mc.vanderbilt.edu/exalt/html/helpIndex.html.

**Figure 1:**
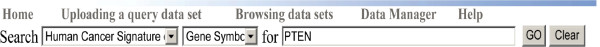
**Multiple search entries**. From any web page interface of the application, users can access the search tool bar and launch a query. The search tool bar supports seven different search fields including gene name, gene symbol, tissue type, experiment type, signature, samples, and multiple genes. Two drop-down menu lists are used for the selections of a database and a database field. The input text box accepts key words from the user.

A search tool bar supports seven different search fields (gene name, gene symbol, tissue type, experiment type, tumor signature, samples, and multiple genes). These fields help build a user-defined query against GEO SigDB or HuCaSigDB. Two drop-down menu lists and one input text box facilitate selections of a database, a search field, and an entry of keywords (Figure 1).

## Illustration 1: Keyword database search

For instance, to identify human cancer signatures containing the tumor suppressor gene *PTEN*, in the first field, a user chooses the database "Human Cancer Signature DB", then selects the database search field as "Gene Symbol", and finally enters the gene symbol, "*PTEN*" in the text box (Figure 1).

**Figure 2:**
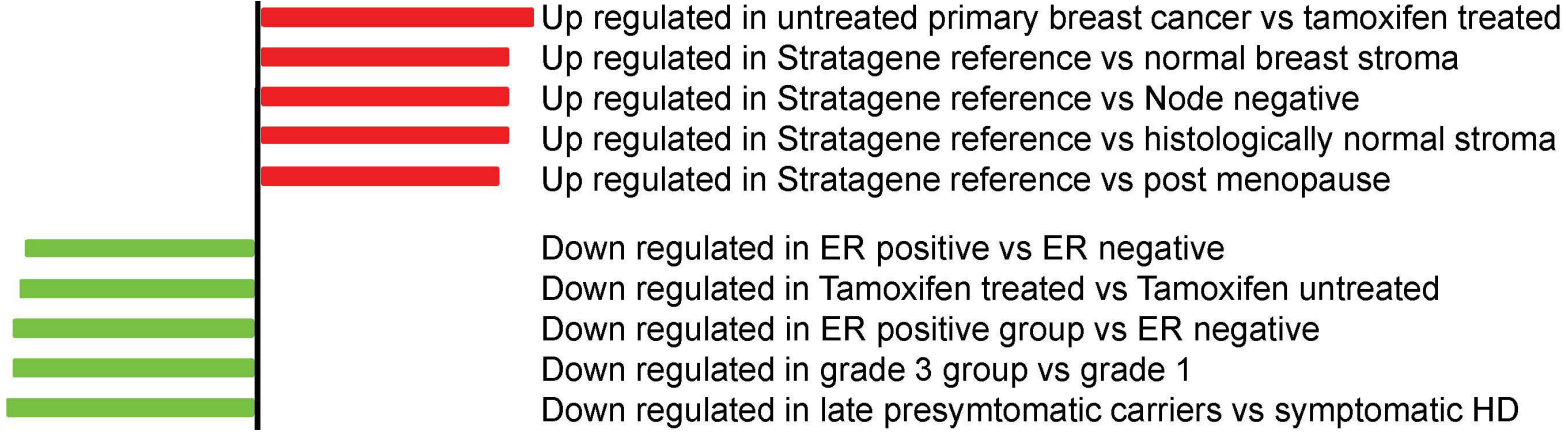
**Meta-gene expression profile across data sets**. The expression profile in HuCaSigDB from a single gene (*PTEN*) query is plotted. The likelihoods of differential gene expression levels are represented by the heights of the bars in units of confidence scores. The differential expression directions in each profile are displayed in two different colors (red for up and green for down).

Once the user has filled out all three fields, the query is submitted using the "GO" button. This particular query returns 548 signature records that are displayed initially in a tabular format. As an option, the user can select to view a color-coded and annotated plot of *PTEN *expression levels among human cancer signatures. Figure 2 illustrates *PTEN *expression levels in ten human cancer signatures selected from the full PTEN profile plot. The likelihoods of differential gene expression levels are represented by the heights of the bars in units of confidence scores. From the *PTEN *profile plot (Figure 2), we observed a common down regulation pattern across multiple datasets for PTEN gene expression among tumor samples with ER+, grade 3, and tamoxifen treatment.

## Illustration 2: Signature homology analysis

**Figure 3:**
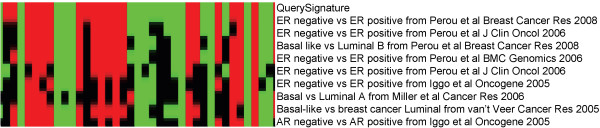
**Meta-heat map of ER related signatures**. The signature names and PubMed sources are listed in the rows, and signature genes are in the columns. The query signature is listed in the first (top) row with matching subject signatures listed after the query signature ranked according to signature similarity scores. The colors in the heat map represent the direction of the differential gene expression within a given signature (red for up, green for down, and black for a missing match), and color intensities reflect the confidence levels of differential expression.

Any signature discovered using the EXALT online program can be used as a query to search for homologous signatures present in the databases. For example, a user may launch a query by selecting a signature from studies of estrogen receptor status (ER negative vs ER positive) in breast cancer [13]. The result of the signature comparisons is illustrated by a meta-heat map in Figure 3. The homologous signatures are listed in the rows, and the signature genes are aligned in alphabetical order in the columns. The query signature is listed in the first (top) row with matching subject signatures listed after the query signature ranked according to signature similarity scores. The colors in the heat map represent the direction of the differential gene expression within a given signature (red for up, green for down, and black for a missing match), and color intensities reflect the confidence levels of differential expression. From this analysis, a cluster of homologous signatures related to the queried signature can be identified. The profile suggests that there might be conserved molecular profiles related to the ER status in breast cancer across multiple human cancer data sets.

## Illustration 3: Gene co-expression analysis

**Figure 4:**
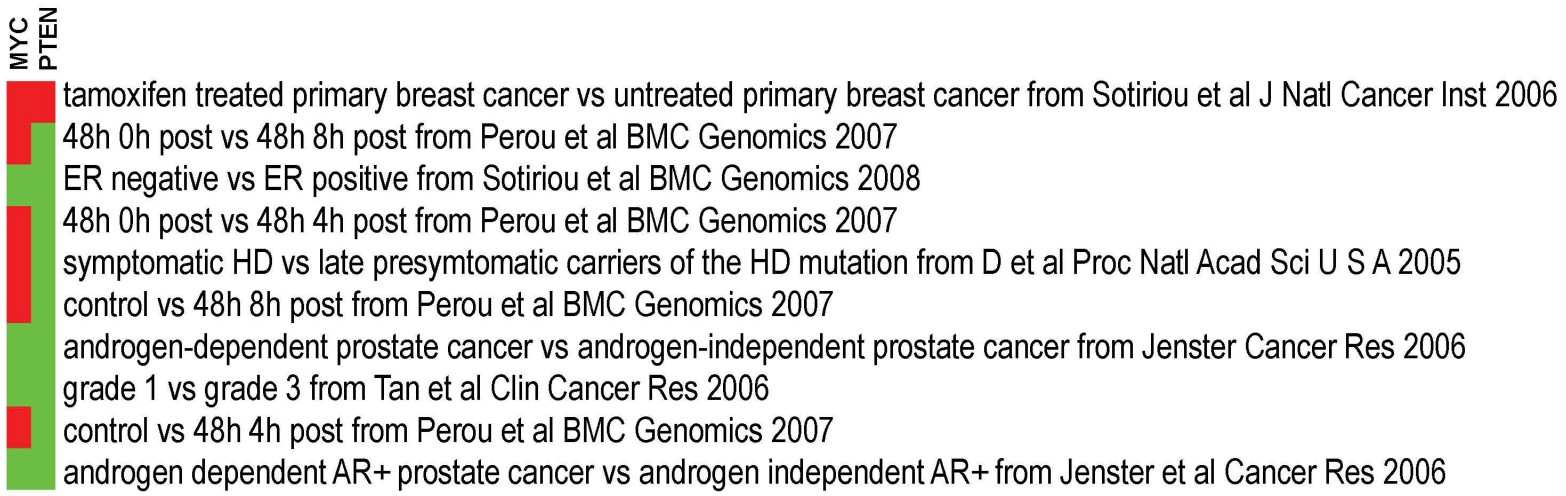
**Co-expression analysis for multiple query genes**. Query genes are labeled in the top of the columns (*MYC *and *PTEN*), and the subject signatures containing two query genes are in the rows represented by a meta-heat map.

Multiple genes from a known pathway or network can be investigated to determine if they co-exist in any signatures. For example, *MYC *is a well-known oncogene, and *PTEN *is a common tumor suppressor gene. Using the EXALT online program we performed an analysis using the human cancer signature database to survey the co-expression profile for these two functionally discordant genes. The returned signatures were ranked by the sum of Q scores for the subject signature triplets from highest (top) to lowest (bottom). Selected results for 10 signature records were plotted as a meta-heat map (Figure 4), which indicated that some cancer signatures had discordant expression of *MYC *and *PTEN *(Figure 4). All search results (Figure 2, 3, and 4), including signature data, plotting source data, and heat map images, can be downloaded from the EXALT online program.

## Discussion

The EXALT online program provides us with a novel way to search and compare publicly available microarray results that can help promote widespread and investigator-driven research on shared data. Our previous report described signature encoding and formatting, the signature extraction process, query data uploading, and algorithms for data set comparison [11]. In this study, we further demonstrated new online features for interrogating signatures (Figure 2, 3, and 4).

The rationale behind the development of the EXALT online program is that it is neither feasible nor beneficial to directly compare raw microarray data. Therefore, we decided to compare summarized microarray results through meta-analysis of gene expression signatures. This strategy enables the EXALT online program to evaluate data similarity across various microarray platforms. Traditional heat map presentation often requires fold changes from the original expression values within a given data set. However, the fold changes are generally not comparable among different experiments. The EXALT online program provides unique output results such as meta-expression plots and meta-heat maps for data from different microarray experiments. These results illustrate differential gene expression changes using differential expression directions, similarity scores, and statistical confidence levels. Thus, we believe that these results allow rapid perusal of relationships between a query signature and entries in a database of other microarray experiments.

We are not aware of any other web-based method currently in use that is capable of searching gene expression signatures by intrinsic similarities among GEO data sets and published microarray studies. Current search engines for gene expression profiles such as GEO and Oncomine are based on microarray meta-data attributes such as sample names, experiment descriptions, or a gene ID. The EXALT online program can perform signature matching through expression profiles extracted from original microarray expression values.

Overlapping gene lists and Venn diagrams have been commonly used to demonstrate relationships among related studies. Gene IDs are the only matching factor in this type analysis, and neither expression direction nor confidence level is considered. This approach provides an easy method to compare gene lists within a study and among published gene lists [14]. However, only limited number of gene lists can be included in this type of analysis, and the extent of overlap among gene lists are often disappointingly low because the lists were generated by heterogeneous analysis strategies.

Gene Expression Atlas (GeneAtlas at http://www.ebi.ac.uk/gxa/) from ArrayExpress database[2] provides an alternative tool to perform gene co-expression analysis. A user defined input contains a query gene list with information about the direction of differential expression and experimental conditions. GeneAtlas displays summarized experimental results across different platforms for identifying strong differentially expressed genes in conditions of interest. A meta-analysis based comparison strategy using pre-computed significant gene lists is also provided by Oncomine, which includes a comprehensive and expertly annotated database of human cancer signatures [12]. This analytical tool enables the search and retrieval of cancer-related expression data or a list of significant genes. However, the comparative meta-profiling to identify shared gene expression signatures across several experiments is limited to meta-data attributes in both GeneAtlas and Oncomine. The meta-data attributes include experiment descriptions, sample information, and probe gene IDs. Differential gene expression data are not comparable across all data sets in the databases. It remains difficult to identify homologous data sets sharing similar expression profiles by comparing microarray results across all studies.

Lamb et al. reported a microrray signature database application using a Gene Set Enrichment Analysis (GSEA), and it is called Connectivity Map (CMAP) [15,16]. The EXALT online program and CMAP have several important differences. At the database level, CMAP signature database has collected only drug-related cancer signatures in 10 cell lines using the same platform and same experimental design. The signature databases used by the EXALT online program include over 28,000 signatures derived from hundreds of different experimental types and many different tissues. CMAP does not require a unified method for the query signature extraction while in the EXALT online program all signatures are generated in the same process. Finally, although both strategies employ a two-group comparison and signed rank-genes as the basis for signature encoding format, biological replicates are not required in CMAP, and no statistical confidence is considered for each ranked-gene. Signature similarity defined in the EXALT online program considers all three key elements (gene ID, direction, and confidence) of gene expression data. Thus, we believe that the EXALT online program offers a more accurate, efficient, and cost-effective way to discover intrinsic similarities and relationships among gene expression profiles.

## Outlook

The EXALT online program is a robust application with a biological question oriented interface and signature search capabilities. The main assets are: (1) the EXALT online program is comprised of two signature databases that have collections of thousands of signatures, (2) the signature annotations are integrated with the NCBI GEO and the PubMed, (3) the multiple mining strategies have been implemented online to allow directly access and analysis, (4) the interfaces are friendly to biologists and clinicians, and knowledge of neither programming language nor statistics tool is required, and finally (5) all signatures are pre-computed and potentially more meaningful to biologists and clinicians.

The next phase of development will include an open web service API to allow other developers to implement modules for deeper analysis of gene expression signature applications. More analysis features and integrations with other data sources will be implemented to extend the application. GEO SigDB will be updated regularly with the latest GEO GDS. More data sets from human cancer studies will be included in the HuCaSigDB, and our ongoing efforts strive to populate this database with human cancer signatures derived from at least 1,000 data sets.

## Conclusions

The EXALT online program is a software application for interrogation of thousands of gene expression profiles in a biologically meaningful contexts. Its architecture supports multiple search entries to query more than 28,000 pre-computed gene expression signatures from GEO and human cancer studies. Meta-expression results are graphically displayed and are straightforward to biologists and clinicians.

## Availability and requirements

•**Project name**: The EXALT online program

•**Project home page**: http://seq.mc.vanderbilt.edu/exalt/

•**Operating systems**: Platform independent

•**Programming language**: ASP.NET

•**Other requirements**: Internet connection

•**License**: Proprietary license. Free for non-commercial purpose

•**Any restriction to use by non-academics**: license needed

## Abbreviations

EXALT: (EXpression signature AnaLysis Tool); API: (Application Programming Interface); GEO: (Gene Expression Omnibus); GDS: (Gene expression Data Set); FDR: (false discovery rate); SigDB: (signature database); HuCaSigDB: (human cancer SigDB); RDBMS: (relational database management system); ASP: (Active Server Page).

## Authors' contributions

YY designed and implemented the database and web application and wrote the manuscript. ALG, YS, and LX helped design and test web application. JF participated in web page implementation. QQ, JW, and JY organized and processed source data sets from human cancer studies. ALG revised the manuscript. All authors read and approved the final manuscript.
